# Long-Range Temporal Correlations of Patients in Minimally Conscious State Modulated by Spinal Cord Stimulation

**DOI:** 10.3389/fphys.2018.01511

**Published:** 2018-10-29

**Authors:** Zhenhu Liang, Jiani Li, Xiaoyu Xia, Yong Wang, Xiaoli Li, Jianghong He, Yang Bai

**Affiliations:** ^1^Institute of Electrical Engineering, Yanshan University, Qinhuangdao, China; ^2^Department of Neurosurgery, PLA Army General Hospital, Beijing, China; ^3^State Key Laboratory of Cognitive Neuroscience and Learning and IDG/McGovern Institute for Brain Research, Beijing Normal University, Beijing, China; ^4^Department of Basic Medical Science, School of Medicine, Hangzhou Normal University, Hangzhou, China

**Keywords:** spinal cord stimulation, minimally conscious state, electroencephalogram, long-range temporal correlations, detrended fluctuation analysis

## Abstract

Spinal cord stimulation (SCS) has been shown to improve the consciousness levels of patients with disorder of consciousness (DOC). However, the underlying mechanisms of SCS remain poorly understood. This study recorded resting-state electroencephalograms (EEG) from 16 patients with minimally conscious state (MCS), before and after SCS, and investigated the mechanisms of SCS on the neuronal dynamics in MCS patients. Detrended fluctuation analysis (DFA), combined with surrogate data method, was employed to measure the long-range temporal correlations (LRTCs) of the EEG signals. A surrogate data method was utilized to acquire the genuine DFA exponents (GDFAE) reflecting the genuine LRTCs of brain activity. We analyzed the GDFAE in four brain regions (frontal, central, posterior, and occipital) at five EEG frequency bands [delta (1–4 Hz), theta (4–8 Hz), alpha (8–13 Hz), beta (13–30 Hz), and gamma (30–45 Hz)]. The GDFAE values ranged from 0.5 to 1, and showed temporal and spatial variation between the pre-SCS and the post-SCS states. We found that the channels with GDFAE spread wider after SCS. This phenomenon may indicate that more cortical areas were engaged in the information integration after SCS. In addition, the GDFAE values increased significantly in the frontal area at delta, theta, and alpha bands after SCS. At the theta band, a significant increase in GDFAE was observed in the occipital area. No significant change was found at beta or gamma bands in any brain region. These findings show that the enhanced LRTCs after SCS occurred primarily at low-frequency bands in the frontal and occipital regions. As the LRTCs reflect the long-range temporal integration of EEG signals, our results indicate that information integration became more “complex” after SCS. We concluded that the brain activities at low-frequency oscillations, particularly in the frontal and occipital regions, were improved by SCS.

## Introduction

Disorder of consciousness (DOC) is a state of prolonged altered consciousness (Eapen et al., 2016). Patients with DOC can be subdivided into minimally conscious state (MCS) and vegetative state (VS) according to their behavioral signs of awareness. Patients with MCS show inconsistent, but discernible signs of consciousness such as command-following or other purposeful behaviors (Giacino et al., 2002).

It has been suggested that spinal cord stimulation (SCS) is an effective brain intervention technique for patients with MCS (Georgiopoulos et al., 2010; Mattogno et al., 2017). SCS has several advantages over deep brain stimulation such as its simplicity, its lesser degree of invasiveness, and its programmable parameters (Bai et al., 2017a). To date, the underlying mechanisms of SCS and its effects on neural responsiveness remain poorly understood (Visocchi et al., 2001; Yamamoto et al., 2012). The effects of SCS in MCS patients can be assessed by several electroencephalography (EEG) measurements (Bai et al., 2017a,b) including entropies, detrended fluctuation analysis (DFA) (D’Rozario et al., 2013), neuronal oscillation coupling (Köster et al., 2014), and integrated information based features (Gallimore, 2015). Entropy is an important measure of both the randomness and the disorder of a dynamic system (Carhart-Harris et al., 2014). Both sample entropy and permutation entropy have been shown to be effective at distinguishing states of consciousness (i.e., wakefulness, deep sevoflurane, and isoflurane anesthesia) (Wang et al., 2014; Liang et al., 2015). Approximate entropy has been found to decrease during the transition from wakefulness to sleep. During wakefulness, it has been shown to be higher in adults than in children (Lee et al., 2013). The coupling strength of narrow-band neuronal oscillations in brain networks has also been shown to be correlated with consciousness (Buzsaki and Draguhn, 2004). Previous studies have suggested that the synchronization of neuronal oscillations is correlated with sensory, motor, and cognitive events (Varela et al., 2001; Jacobs and Kahana, 2010). Based on the synchronization phenomenon in neuronal activities, many measures, such as phase-amplitude coupling, coherence, and phase synchronization, have been proposed for consciousness assessment (Liang et al., 2016; Bai et al., 2017b; Pal et al., 2017). Our previous study has suggested that bicoherence, global synchronization (Bai et al., 2017a), as well as global and local networks (Bai et al., 2017b), can be altered by SCS with 70 Hz in MCS patients.

In addition to the features mentioned above, it has also been hypothesized that consciousness emerges from the critical state of brain activity. The hierarchy of long-range temporal correlations (LRTCs) implies the long-term memory of a brain system (Zhang et al., 2018). In the temporo-spatial theory of consciousness (TTC), LRTCs are postulated to be a core mechanism of consciousness (Northoff and Huang, 2017). They exist in “scale-free” systems based on fractal theory (Richard et al., 2012). “Scale-free” systems involve two phenomena: self-similarity and self-affinity. Self-similarity means that a small part of the fractal structure is similar to the entire structure, while self-affinity indicates that the properties of a fractal scale are different along various dimensions (Mandelbrot, 1999). These two phenomena cannot be easily characterized by their respective means and standard deviations (Eke et al., 2000). Therefore, “scale-free” systems can only be measured by the power–law function, with only a mathematical function, without a typical scale (Richard et al., 2012). Accordingly, DFA is widely used to analyze the scale-free time series (Palva et al., 2013) with three correlation properties of the signals: (1) uncorrelated scaling (DFA > 1), correlated scaling (0.5 < DFA < 1), and anti-correlated scaling (0 < DFA < 0.5) (Hu et al., 2001). The correlated scaling (0.5 < DFA < 1), also known as LRTC, indicates long-range temporal dependency characteristics of the time series. Many studies have investigated the LRTCs of the neural signals under general anesthesia (Krzeminski et al., 2017; Zhang et al., 2018), during sleep (Tagliazucchi et al., 2013; Allegrini et al., 2015), and in states of self-consciousness (Huang et al., 2016). These studies have found that the brain dynamics present robust LRTCs during conscious states. Whereas, the LRTCs are disrupted during unconscious states. Given that the LRTCs can reflect complex neural information processing, we hypothesize that the effect of SCS can be measured by LRTCs in the long-range temporal dependency framework. Based on this hypothesis, this study combines the DFA and the surrogate data method to measure the genuine LRTCs. We then investigate the temporo-spatial changes of genuine LRTCs of the EEG signals in MCS patients during SCS.

## Materials and Methods

### Subjects

The eligibility criteria for the subjects were: (1) patients had been diagnosed as MCS by the JFK Coma Recovery Scale-Revised (CRS-R) (Kalmar and Giacino, 2005); (2) patients were in stable clinical states; and (3) there were no confounding complications (e.g., infections). This study was approved by the ethics committee of the PLA Army General Hospital. Informed consent to participate in the study was obtained from legal representatives of the patients.

### Data Recording and Preprocessing

EEG recording was conducted at least 3 weeks after the SCS surgical procedure. Treatments other than SCS that could modify neural excitability were avoided during EEG recording. The SCS stimulation protocol follows our previous study (Bai et al., 2017a), summarized as follows: The SCS stimulator (Prime Advanced, Medtronic Inc., Minneapolis, MN, United States) was placed under the anterior chest wall. The stimulation frequency was set at 70 Hz, based on clinical experience. The amplitude and duration of the pulses were 3 V and 210 μs, respectively. The stimulator produced a periodic voltage difference between the two stimulation electrodes, with a duration of 20 min. The electrodes sent pulses to stimulate the specific level of the spinal cord. All subjects were in the supine position, and in a wakeful state, throughout the study. If the subjects showed to be in a sleep state (i.e., prolonged eye closure or sleep waveforms, such as spindles or K-complex waves appearing in the EEG), the study would be paused. The JFK CRS-R arousal facilitation protocol would then be performed to arouse the subjects.

**FIGURE 1:**
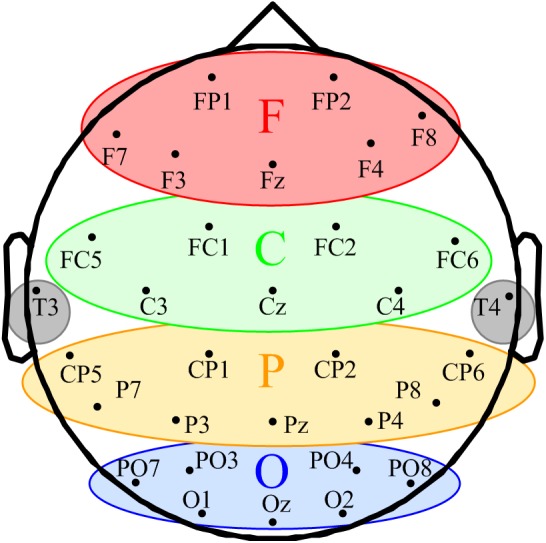
Schematic of space participation of five regions for 32-channel EEG. The seven channels in the red ellipse constituted the frontal region (F). The seven channels in the green ellipse were divided into the central region (C). The yellow ellipse comprised nine channels represents the parietal region (P). The blue ellipse comprised nine channels represents the occipital region (O). T3 and T4 in the gray circles were classified into the temporal region.

A 10 min EEG was recorded both before and after SCS. A 32-channel EEG cap (BrainAmp 64 MRplus, Brain Products, Germany) with Ag/AgCl electrodes was used for EEG recording. Electrode positioning was based on the international 10–20 electrode placement system, as shown in Figure 1. The sampling rate of the system was 1 kHz. A conductive EEG gel was used to remove cutin and oil from subjects’ scalps. The electrode–skin impedance of subjects was decreased to less than 5 kΩ before the recording.

The EEG signal preprocessing was conducted with EEGLAB (version 12.0.2.5b) in a MATLAB environment (Version, 2014a, MathWorks Inc.; Natick, MA, United States). Continuous data segments with significant noise were rejected based on visual inspection. The 50 Hz power frequency artifact was removed by the notch filter. The EEG data was filtered into 1–45 Hz and downsampled to 100 Hz using symmetric finite impulse response filters (MATLAB function firls.m) and the MATLAB function *resample.m*, respectively. The relevant components of the artifacts, such as eye movements and muscle activities, were identified and removed by the independent component analysis function in EEGLAB. After pre-processing, the EEG signals were divided into five frequency bands: delta (1–4 Hz), theta (4–8 Hz), alpha (8–13 Hz), beta (13–30 Hz), and gamma (30–45 Hz).

### Genuine DFA Exponent (GDFAE)

**FIGURE 2:**
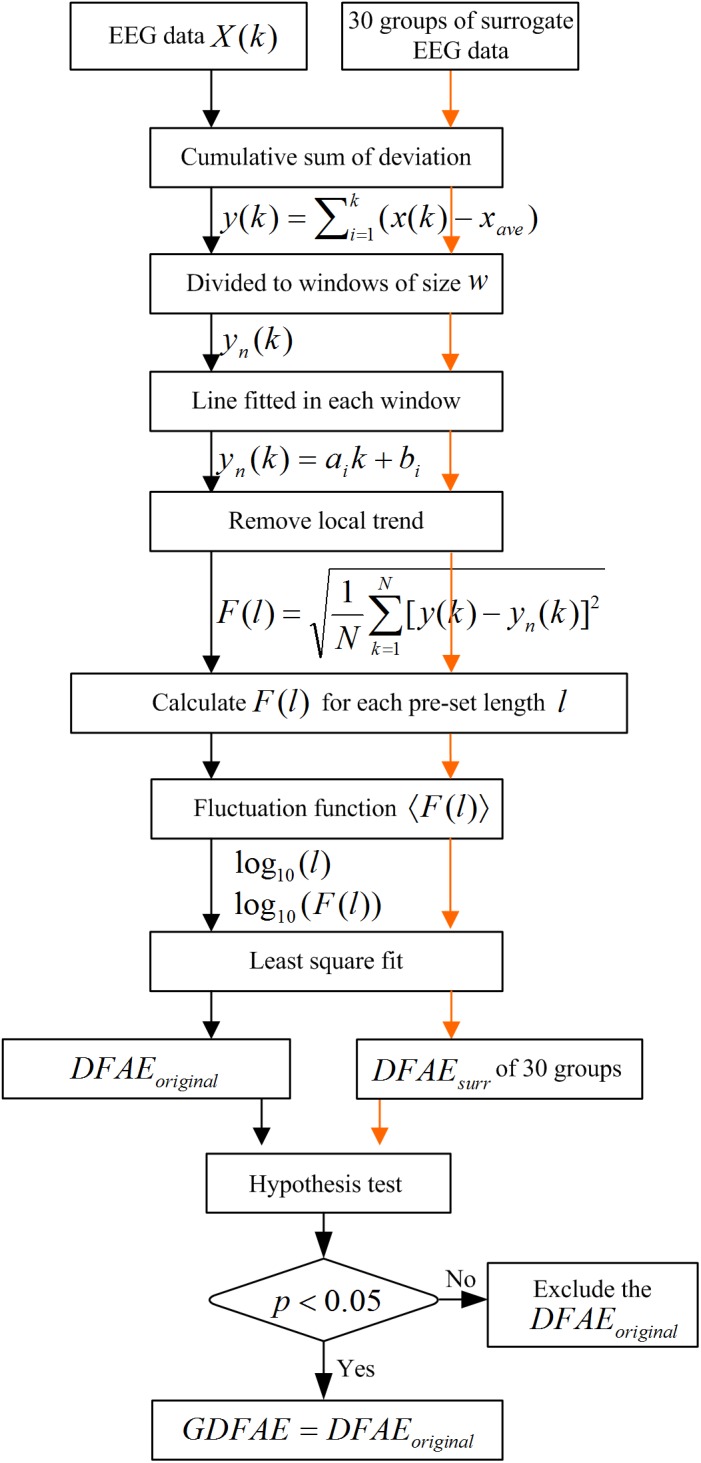
Flow diagram for GDFAE calculation.

**FIGURE 3:**
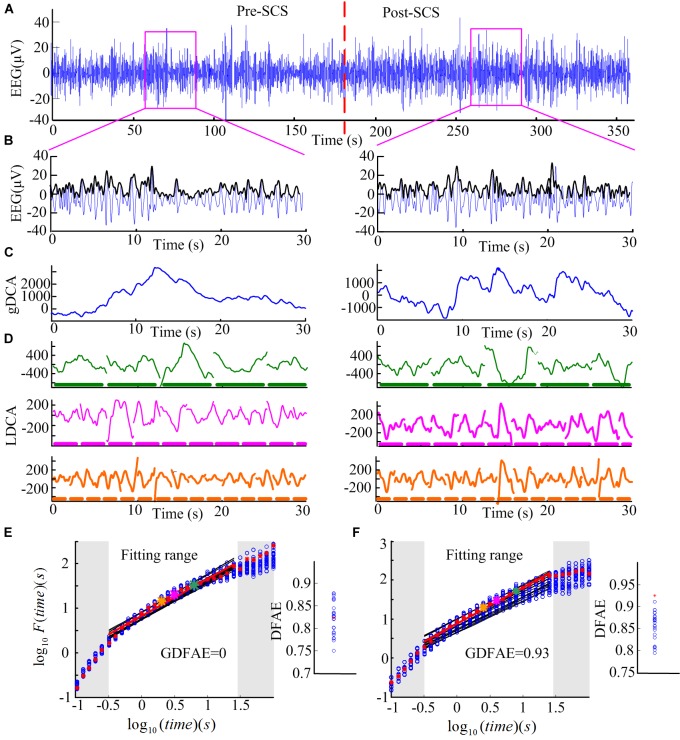
Step-by-step illustration of DFAE calculation. **(A)** Delta band preprocessed EEG signals totaling 6 min (3 min before SCS and 3 min after SCS) from participant No. 2 at channel FP2. The red dash line denotes the SCS. **(B)** Delta band EEG signals of 30 s were extracted from the two states (blue line) and amplitude envelope (black line). **(C)** Detrended cumulative amplitude, without the global linear trend, of both states. **(D)** LDCA of time window of length 6.3 s (top), 3.2 s (middle), and 2.0 s (bottom); the thick dash line parallel to the timeline represents the time windows, the length of each piece on behalf of the length of the time window–green for 6.3 s, pink for 3.2 s, and orange for 2.0 s. **(E)** The fitting of the logarithmic fluctuation function for both real EEG signals and surrogate EEG data performed for the pre-SCS state; red asterisks and hollow blue circles represent the real and the surrogate EEG respectively. The big asterisks correspond to the same color time window in panel **(D)**. The red and black lines are the linear regression fitted lines. To obtain a reliable exponent, the log times (windows) in the gray area were excluded from the linear regression. The windows which are too narrow (in the lower left corner) exhibit inherently steeper scaling, whereas windows too large (i.e., those in the upper right corner) may induce the lacking of data for reliably estimating the variability. The right part is the corresponding slope (*DFAE*) value of the function. The blue circles represent DFAE_surr_ and the red asterisk represents DFAE_original_. This significance test shows that this channel, in this state, is a spurious DFA channel. Thus, the GDFAE = 0. **(F)** The fitting of the logarithmic fluctuation function for the post-SCS state. Similar to panel **(E)**, but the significance test showed that it is a genuine DFA channel in this state (GDFAE = *DFAE_original_* = 0.9251).

We combined the DFA and the surrogate data method to evaluate the effect of SCS at different oscillation bands. DFA is a classic measure of the LRTCs of EEG activity (Peng et al., 1995). It quantifies the fluctuations of a time series based on the power-law method. DFA can systematically remove the trend of each order from the data. Thus, it has a robust effect against noise. The surrogate data method has been widely applied to constrain spurious detection in non-linear analysis (Dolan and Spano, 2001). In this study, surrogate data tests were utilized to acquire the GDFAEto reflect the genuine LRTCs of brain activities. The flow chart of the algorithm and the step-by-step results are shown in Figures 2, 3, respectively.

The GDFAE can be obtained in three steps. The detailed process is as follows:

Step 1: Calculating the original DFA exponent of the EEG data.
(1) Remove the mean value *x_ave_* of the amplitude envelope of the EEG time series *x* (*t*) to obtain a series independent of the global linear trend. The cumulative sum of the detrended amplitude *y* (*k*) is calculated as: *N* indicates the length of *x* (*t*). The new time series *y* (*k*) is called the global detrended cumulative amplitude (gDCA). To clarify the DFA procedure, sequences of 30 s were extracted and calculated for the presentation. The extracted data of 30 s from both states (pink rectangle in Figure 3A) and their amplitude envelopes, are shown in Figure 3B. The gDCA for both states (pre- and post-SCS) were thus obtained (Figure 3C).
(2) The gDCA *y* (*k*) was divided into several non-overlapping subsequences of length *l*. Each subsequence corresponded to a time window, and the length of the time windows was defined as eight times larger than the filter order and eight times smaller than the recording length (Hardstone et al., 2012). The least squares method (LSM) was applied to fit the trend of each time window as a straight line *y_n_* (*k*) = *a_i_k* + *b_i_*, where *a_i_* and *b_i_* were the undetermined coefficients. In each time window, *y_n_* (*k*) was subtracted from the primary *y* (*k*) to remove the local trend. The fluctuation function *F* (*l*) was quantified as a square deviation function, that is, the average root mean square of the locally detrended cumulative amplitude (LDCA) as follows:

(1) $$F(l)=\sqrt{\frac{1}{N}\overset{N}{\underset{k=1}{\Sigma }}{\left[y(k)-y_{n}\left(k\right)\right]}^{2}}$$

All fluctuation functions *F* (*l*) of the time windows of different length *l* were calculated, where *l* is defined equidistantly on a logarithmic scale. Figure 3D shows the LDCA of a time window with lengths of 6.3 s (top), 3.2 s (middle), and 2.0 s (bottom).
(3) The fluctuation function *F* (*l*) and window length *l* were transformed into a logarithmic coordinate, and LSM was used to estimate the linear trend and slope α of the fitted line. α is also commonly referred to as the Hurst scaling exponent *H* (Colombo et al., 2016) or the DFA exponent (Krzeminski et al., 2017). The DFA exponent of the original EEG was abbreviated as *DFAE_original_*. In Figures 3E,F, the green, pink, and orange asterisks correspond to the time windows of 6.3, 3.2, and 2.0 s in (D), respectively.
Step 2: Calculating the surrogate data set for the original EEG signals.
First, the real recorded EEG signals *x*(*n*) were transformed into the frequency domain *X* (*k*) by the discrete Fourier transform as follows: $X(k)=\Sigma_{n=0}^{N-1}x(n)e^{-j2\pi kn/N}$. Second, a uniform random sequence *v* (*k*) was produced. The phase spectra of *X* (*k*) was replaced by the random sequence *v* (*k*) with the amplitude spectra of *X* (*k*) remaining unchanged, that is, *S* (*k*) = |*X* (*k*)| *e*^*jv* (*k*)^. The existing spurious temporal correlations in the signal were replaced, but the spectrum shape was preserved. Finally, the surrogate data were obtained through the inverse discrete Fourier transform $s(n)=\frac{1}{N}\Sigma_{k=0}^{N-1}S(k)e^{j2\pi kn/N}$ (Schreiber and Schmitz, 1996, 2000). For the frequency band of each channel, 30 groups of surrogate data were produced.
Step 3: Obtaining the GDFAE at each frequency band.
The surrogate DFA exponents were calculated for each band and termed *DFAE_surr_*. The Wilcoxon signed-rank test (*signrank.m*) was applied to eliminate any spurious DFA exponents. The *DFAE_surr_* was tested with *DFAE_original_*. Their difference sequence was tested with the hypothesis that the element in the sequence comes from a distribution with a median of 0. The significance threshold was set to the 95% confidence level. If the null hypothesis was rejected at the 5% level (*p* < 0.05 and *h* = 1), the *DFAE_original_* would be different from the distribution of 30. Thus, the *DFAE_original_* was the exact GDFAE. Otherwise, the *DFAE_original_* would have been considered a spurious LRTC value. This can also be described as follows:

(2) $$GDFAE=\{\begin{array}{c} {DFAE}_{original}:h=1,p<0.05\\ null:otherwise \end{array}$$

The GDFAE describes the robust correlation characteristics of a time series (Colombo et al., 2016). When *GDFAE* =0.5, the signals are white noise with no autocorrelation. When *GDFAE* =1, the signals are 1/f noise. When 0.5 < *GDFAE* < 1, the signals are temporally anti-correlated. When 0.5 < *GDFAE* < 1, the recorded EEG signals are positive temporal correlations, that is, LRTCs. Thus, the neural activity at a given time could potentially have been influenced by the neural activities which had occurred several minutes before (Maxim et al., 2005; He, 2011).
Figures 3E,F present the surrogate analysis of two EEG periods. The logarithmic function and the corresponding window length for the real EEG (red asterisks) and the surrogate data (blue circle) are fitted in the red and black lines, respectively. The right part of Figure 3E shows the distribution of the DFAE for the real EEG (*DFAE_original_*) and the surrogate EEG (*DFAE_surr_*). *DFAE_original_* is not distinctly different from the distribution of *DFAE_surr_*. This indicates that this EEG segment lacks genuine LRTCs (GDFAE = 0). Conversely, Figure 3F shows the GDFAE, which has genuine LRTCs.

In the DFA measurement, selecting the size of the windows for linear fitting is crucial (Kantelhardt et al., 2001). If the length of each window is too large, there will not be enough windows for calculation, and the results may be inaccurate. Although this problem can be alleviated by overlapping windows, window length must be no larger than 10% of the signal length (Hardstone et al., 2012). Conversely, if the length of each window is too short, more scaling will be observed in the fluctuation function. With the increasing length of the windows, the scaling behavior will converge asymptotically. The lengths of the windows (*l*) are represented equidistantly on a logarithmic scale (see in Figure 3E). The latter, because of the fluctuation functions [*F* (*l*)] and the corresponding *l*, will be transformed into a logarithmic coordinate to obtain slope α. Linear fitting was performed only for windows in which the fluctuation function depicted a straight line to get a reliable exponent α. To eliminate the edge effect, we used an automated process (Fell et al., 2000). First, a histogram of the first derivative for each pair of the adjacent fluctuation data was plotted. Second, the majority of the derivative values were grouped together. Finally, the 90th percentile value of the distribution was chosen as the threshold for fitting (Krzeminski et al., 2017).

### Statistical Analysis

This study evaluated the changes in LRTCs after SCS in MCS patients. We analyzed the GDFAE in different brain regions, at different frequency bands, before and after SCS. Considering the multi-factor effects, a three-way repeated analysis of variance (three-way ANOVA; *anovan.m*) was applied to analyze the interactions and the main effects of the three factors: (I) brain states (two levels: pre- and post- SCS), (II) frequency bands (five levels: delta, theta, alpha, beta, and gamma), and (III) brain regions (four levels: frontal, central, parietal, and occipital). After obtaining the interactions and the main effects of the three factors, a *post hoc* test with the Bonferroni correction was used to determine the significant difference of the GDFAE values between the two states in each brain region, at each frequency band.

## Results

According to the eligibility criteria, 16 MCS patients were enrolled in this study. Their demographic data and clinical diagnoses are presented in Table 1. According to our previous study, the EEG channels were divided into five regions. The frontal region (F): FP1, FP2, Fz, F3, F4, F7, and F8; the central region (C): FC1, FC2, FC5, FC6, Cz, C3, and C4; the parietal region (P): CP1, CP2, CP5, CP6, Pz, P3, P4, P7, and P8 (Figure 1); the occipital region (O): PO3, PO4, PO7, PO8, Oz, O1, and O2; the temporal region: T3 and T4. Since the temporal region only included two channels, we excluded the temporal region from the analysis of the changes in LRTCs.

**Table 1:** Patient demographics.

Subject	CRS-R	Etiology	Post_injury (months)
1	8	Hemorrhage	18
2	9	Hemorrhage	5
3	10	Traumatism	9
4	10	Hemorrhage	12
5	7	Ischemia-hypoxia	4
6	8	Ischemia-hypoxia	3
7	7	Hemorrhage	10
8	9	Traumatism	3
9	7	Hemorrhage	5
10	7	Hemorrhage	4
11	7	Ischemia-hypoxia	3
12	7	Hemorrhage	11
13	10	Ischemia-hypoxia	4
14	8	Traumatism	13
15	8	Ischemia-hypoxia	4
16	9	Hemorrhage	6


*CRS-R, coma recovery scale-revised.*

**FIGURE 4:**
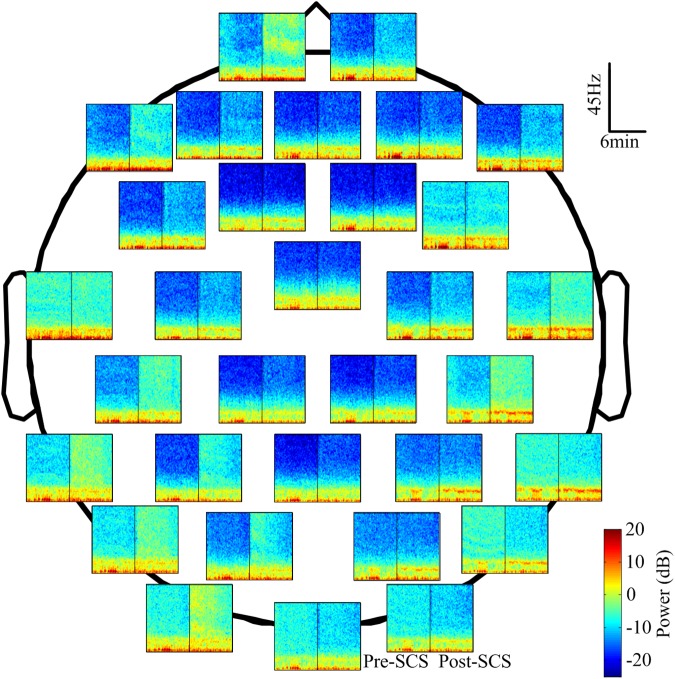
The power spectrum of 32 channels in the corresponding position of one participant (No. 1 in Table 1). The spectrum was computed with the short-time Fourier transform with a Hamming window. The blue represents the lower power and the dark red represents the higher power. The black line in each spectrum block represents the interposition of the SCS.

The EEG spectra of all channels were calculated to reveal the changes in EEG oscillation after SCS. To illustrate this, Figure 4 shows the power spectra of one subject in which the power was concentrated at the delta and theta bands. The spectra of the different channels was diverse. The power of the high frequency bands (beta and gamma) increased after SCS in most channels such as FP1, FP2, and F7. In some of the channels, such as FC1, FC2, and CZ, it did not. The color bar was set from -25 to 20 dB to highlight the differences between the two states (pre- and post-SCS). The maximum power differential between the pre-SCS and the post-SCS state was 15 dB.

**FIGURE 5:**
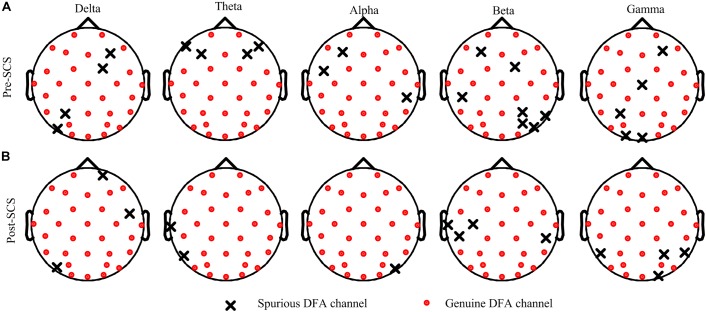
Surrogate data results for one participant (No. 7 in Table 1). **(A,B)** are the spatial distribution of the genuine and spurious DFA channels at five frequency bands, at pre- and post-SCS states. The solid red circle represents the genuine DFA channel and the black crosses represent the spurious DFA channel.

Then, the *DFAE_original_* and *DFAE_surr_* of each channel, at each frequency band, were calculated to obtain the GDFAE. If the *DFAE_original_* deviated from the distribution of *DFAE_surr_*, this indicated that the EEG data in this state had genuine LRTCs. Figure 5 shows the distribution of the genuine and spurious LRTCs at each frequency band for one subject, both before and after SCS. The quantity of genuine LRTCs channels increased after SCS. However, the significant increases in spatial distribution were not consistent for each frequency band. The proportions of genuine LRTC channels at each frequency band are presented in Table 2. The results indicate that at all five frequency bands, the spatial distribution of the genuine LRTCs broadened after SCS.

**Table 2:** The proportion of the significant GDFAE in pre- and post-SCS stage, at different frequency bands [Median (min–max)].

	Delta	Theta	Alpha	Beta	Gamma
Pre-SCS	0.91 (0.84–1.00)	0.87 (0.75–0.97)	0.92 (0.91–0.94)	0.92 (0.78–1.00)	0.88 (0.75–1.00)
Post-SCS	0.94 (0.91–1.00)	0.93 (0.72–1.00)	0.93 (0.81–1.00)	0.93 (0.81–1.00)	0.93 (0.88–1.00)


**FIGURE 6:**
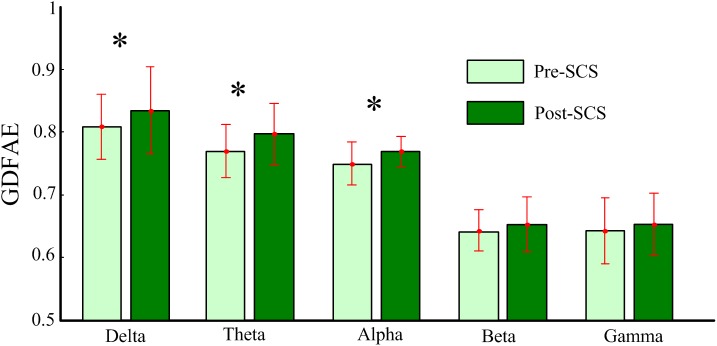
Descriptive statistics for the averaged GDFAE for all patients. This includes the bar (mean) and the error bar (standard deviation) of the averaged GDFAE at five frequency bands, at pre- and post-SCS states. The asterisk denotes a *p*-value less than 0.05 in multiple comparison test, with Bonferroni correction.

To explore how SCS modulated the LRTCs at different frequency bands, the averaged GDFAE values of the 32 channels for each subject, at each neural oscillation band (delta, theta, alpha, beta, and gamma), were calculated. The descriptive statistics for the averaged GDFAE in the pre-SCS and post-SCS states are presented in Figure 6. The mean and standard deviation values in Figure 6 are listed in Table 3. The value of the averaged exponents in both states ranged from 0.5 to 1. The GDFAE values at the delta, theta, and alpha bands were larger than those at the high-frequency bands (i.e., beta and gamma). The mean values of the averaged GDFAE increased significantly in the post-SCS state compared to those from the pre-SCS state, especially at the delta (*p* = 0.039), theta (*p* = 0.021), and alpha (*p* = 0.032) bands. The results of the multiple comparison test are shown in Figure 6. Furthermore, the standard deviation of the GDFAE was smaller at the alpha band than it was at the other frequency bands.

**Table 3:** Descriptive statistics for average GDFAE of each participant in pre- and post-SCS stages, at different frequency bands (mean ± SD).

	Delta	Theta	Alpha	Beta	Gamma
Pre-SCS	0.809 ± 0.052	0.770 ± 0.042	0.750 ± 0.034	0.643 ± 0.033	0.642 ± 0.052
Post-SCS	0.835 ± 0.070	0.797 ± 0.049	0.769 ± 0.024	0.653 ± 0.044	0.653 ± 0.050


**FIGURE 7:**
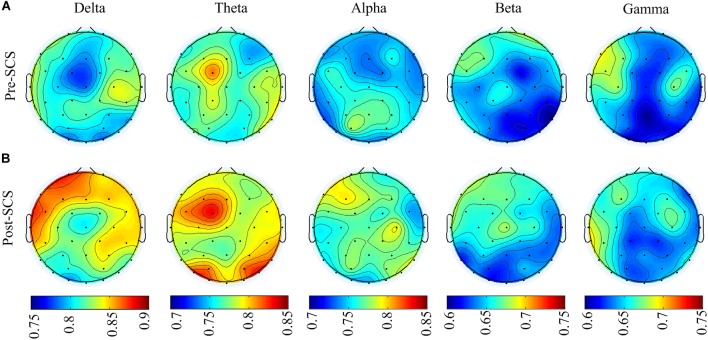
**(A)** Spatial distribution topographic of the non-zero GDFAE at each frequency band in pre-SCS state. **(B)** Topographic of the post-SCS state.

To analyze the effect of SCS on different spatial regions, the GDFAE values of the five frequency bands were analyzed (including four brain regions). The *topoplot* function in EEGLAB was applied to obtain a two-dimensional topographical map of the GDFAE value. The spatial distributions of the averaged non-zero GDFAE values of the 16 subjects at each frequency band are presented in Figure 7. Non-zero GDFAE values ranged from 0.5 to 1, indicating that the EEG dynamics were presented in the LRTCs. Moreover, the LRTCs were stronger in the post-SCS state at the delta, theta, and alpha frequency bands (Figure 7). Lastly, the spatial distribution showed an increase in the frontal and occipital regions.

**FIGURE 8:**
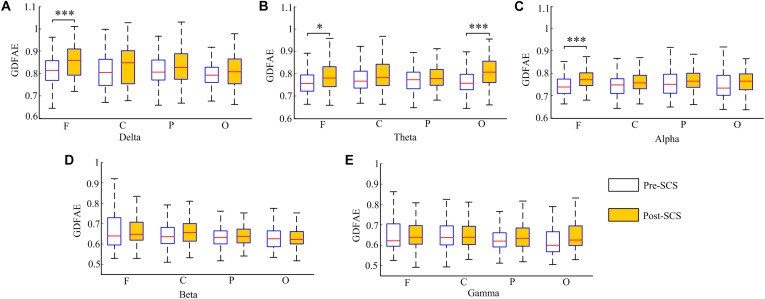
Box plot of the GDFAE for all 16 patients. Each graphic includes both states of all four regions at one frequency band. The symbol “^∗∗∗^” denotes a *p*-value less than 0.001, “^∗∗^” denotes a *p*-value less than 0.01, and “^∗^” denotes a *p*-value less than 0.05.

The GDFAE statistics for all 16 patients are shown as box plots in Figure 8. Three-way ANOVA and a multiple comparison test (*multcompare.m*) with the Bonferroni correction were used to test significance. The three-way ANOVA analysis showed the interactions and the main effects of the three factors [state (I), frequency band (II), and brain region (III)]. The interactions of I^∗^II and I^∗^III were both significant (*p* < 0.001 and *p* < 0.05, respectively). This indicated that the changes in the GDFAE after SCS were related to the brain regions and frequency bands. In other words, the variation between the pre- and the post-SCS GDFAE in different brain regions or at different frequency bands varied in degree. The main effects of factor I were significant (*p* < 0.001), indicating that the GDFAE values in pre- and post-SCS states were significantly different. A multiple comparison test analyzed the difference between the pre- and post-SCS for the different brain regions and frequency bands. We used the Bonferroni correction for the number of frequency bands and brain regions to account for the repetition of testing. Figure 8 shows that the GDFAE significantly increased at delta, theta and alpha bands after SCS. At the delta band, the significant increase was in the frontal region (*p* < 0.001). At the theta band, a significant increase was found in the frontal (*p* < 0.05) and occipital regions (*p* < 0.001). A significant increase at the alpha band was also observed in the frontal region (*p* < 0.001). However, no significant increase or decrease was found at the beta or gamma bands in any brain region. The indices in the box plots were not normally distributed (Lilliefors test). Therefore, all indices were expressed as medians (min–max), and are listed in Table 4. The *p*-values of the significance test for the GDFAE statistics are shown in Table 5.

**Table 4:** Descriptive statistics for GDFAE in different brain regions in pre- and post-SCS stages, at different frequency bands [Median (min–max)].

	Delta	Theta	Alpha	Beta	Gamma
Pre-F	0.81 (0.77–0.86)	0.76 (0.72–0.80)	0.74 (0.71–0.77)	0.68 (0.60–0.73)	0.62 (0.60–0.70)
Post-F	0.86 (0.79–0.91)	0.78 (0.74–0.83)	0.77 (0.75–0.80)	0.65 (0.62–0.71)	0.64 (0.60–0.70)
Pre-C	0.81 (0.75–0.85)	0.77 (0.74–0.81)	0.75 (0.71–0.78)	0.63 (0.60–0.68)	0.64 (0.60–0.69)
Post-C	0.83 (0.75–0.90)	0.78 (0.75–0.84)	0.76 (0.73–0.79)	0.66 (0.61–0.70)	0.63 (0.60–0.70)
Pre-P	0.81 (0.77–0.86)	0.77 (0.73–0.81)	0.75 (0.71–0.80)	0.60 (0.60–0.68)	0.62 (0.60–0.66)
Post-P	0.83 (0.74–0.89)	0.78 (0.75–0.82)	0.77 (0.74–0.80)	0.64 (0.61–0.67)	0.63 (0.60–0.69)
Pre-O	0.79 (0.76–0.83)	0.76 (0.73–0.80)	0.73 (0.70–0.79)	0.62 (0.59–0.66)	0.60 (0.57–0.67)
Post-O	0.81 (0.76–0.87)	0.81 (0.76–0.86)	0.77 (0.73–0.80)	0.62 (0.60–0.66)	0.63 (0.60–0.70)


*Pre-F, pre-SCS stage in frontal region; Post-F, post-SCS stage in frontal region; Pre-C, pre-SCS stage in central region; Post-C, post-SCS stage in central region; Pre-P, pre-SCS stage in parietal region; Post-P, post-SCS stage in parietal region; Pre-O, pre-SCS stage in occipital region; Post-O, post-SCS stage in occipital region.*

**Table 5:** *p*-Value of the significance test for the GDFAE statistics in Figure 8.

	Delta	Theta	Alpha	Beta	Gamma
F	1.108e-04	0.035	3.149e-05	0.782	1.000
C	0.100	0.275	0.461	0.996	0.937
P	0.442	0.758	0.761	1.000	0.215
O	0.756	2.469e-07	0.178	0.995	0.929


## Discussion

This study investigated the dynamic brain activity changes in pre- and post-SCS state of MCS patients. The GDFAE was employed to assess the LRTCs of EEG signals, recorded from 16 MCS patients. We analyzed the GDFAE changes in different brain regions, at different frequency bands. The results indicated that the LRTCs in the post-SCS state were more enhanced than those in the pre-SCS state, in some regions and frequency bands. The main findings are as follows: (i) The proportion of the non-zero GDFAE in the post-SCS state was higher than that of the pre-SCS state [i.e., the quantity of channels with GDFAE increased at all frequency bands in the post-SCS state (Table 2)]. This indicated that the long-range temporal integration became more widespread after SCS. (ii) The GDFAE value increased after SCS, especially at the lower frequency bands (delta, theta, and alpha). The increased GDFAE suggested that the LRTCs in this neural oscillation had become stronger after SCS. (iii) The LRTCs of the frontal region significantly increased in the post-SCS state at the delta, theta, and alpha frequency bands. The occipital region also showed a significant increase at the alpha band (Figure 8 and Table 5). The dominant enhancement of LRTCs in the frontal region and at the alpha frequency band could provide more evidence for the potential mechanisms of SCS in modulating the brain activities of MCS patients.

This study utilized the DFA method. In addition to DFA, other measures and theories have been proposed to analyze the EEG signals. Among them, entropy is an important non-linear method. Both the fluctuation of the entropy and the DFA exponents of the EEG signals can indicate the complexity of the brain system (Lee et al., 2004; Morabito et al., 2012). However, when applied to pathologic signals, DFA is recommended over entropy due to its capability to discriminate and predict the occurrence of a pathological state (e.g., epileptic seizure) (Cirugeda-Roldán et al., 2012).

According to the DFA methodology, the LRTCs in EEG signals are related to conscious behaviors. The larger fluctuation corresponded to the strengthening of the long-term memory of the underlying neural processes. When the LRTCs are weakened, the information integration tends to break down or decline (Thiery et al., 2018). For example, Krzeminski et al. (2017) found a breakdown in LRTCs at the alpha frequency band during general anesthesia. The DFA exponents decreased from the value associated with wakeful state to that of general anesthesia. The authors hypothesized that the brain activity exhibited robust LRTCs, and thus could be disrupted during general anesthesia. A similar phenomenon has also been observed during sleep with a decrease in long-term memory in the default mode and attention networks (Tagliazucchi et al., 2013). Insomnia has also been found to be related to LRTCs. Individuals who experienced worse sleep quality tended to have stronger LRTCs during wakefulness (Colombo et al., 2016). Furthermore, the LRTCs could be controlled by engaging an intrinsic neuroregulation through a closed-loop neuro-feedback stimulation–the LRTCs were found to be stronger during stimulation (Zhigalov et al., 2016). Based on the studies mentioned above, the fluctuations in the LRTCs were related to neural behaviors. Thus, in our results, the increased LRTCs may indicate that a more “complex” cortical information integration is restored after the SCS. From the perspective of complexity, various studies have shown that loss of consciousness is correlated with a decrease in complexity, such as in general anesthesia (Alonso et al., 2014; Liang et al., 2015; Schartner et al., 2015; Hudetz et al., 2016) and sleep (Priesemann et al., 2013). We concluded that the SCS increases brain dynamics in MCS patients.

After SCS, not only did the value of GDFAE increase, but the non-zero (genuine) proportion of DFA increased as well. To obtain the genuine proportion of DFA, the DFA was combined with the surrogate data method. The crux of the surrogate data method is to eliminate the non-linear correlation by reconstructing the power spectrum while maintaining the same linear feature. The advantage of surrogate data is that it preserves the linear stochastic structure and the amplitude distribution of the original series (Lucio et al., 2012). The two most commonly used algorithms for generating surrogate data are the amplitude-adjusted Fourier transform (AAFT) and the iterated AAFT (Rath and Monetti, 2008). The AAFT is considered a robust surrogate data-generated method and requires less computation than other methods (Pritchard et al., 2010). Using the AAFT, a GDFAE can be achieved that can reflect genuine LRTCs. If the DFAE is not genuine, there will be no LRTCs. Our results showed that the genuine LRTCs are spread more widely after the SCS. Also, more cortical areas were engaged in information integration after the SCS.

The results of this study have also revealed that most of the increased LRTCs occurred at the delta, theta, and alpha frequency bands. This suggests a frequency specificity for the LRTCs in MCS patients. Several other pathological investigations have found that the frequency-specific of LRTCs, such as the theta-band LRTCs, decreases in patients with major depression disorder (Linkenkaer-Hansen et al., 2005). Alpha-band LRTCs have also been shown to decrease in patients with Alzheimer’s disease (Montez et al., 2009). In propofol-induced unconsciousness, no frequency-specific LRTCs were observed (Krzeminski et al., 2017). It has been speculated that cross-frequency interactions play a key role in this phenomenon. The temporal dynamic changes at one frequency band may affect other bands, given that the lower frequency oscillations (i.e., delta, theta, and alpha bands) in MCS patients are typically different from those of healthy subjects (Fingelkurts et al., 2012).

The spatial statistics showed that the GDFAE significantly increased in the frontal region at the delta (*p* < 0.001), theta (*p* < 0.05), and alpha (*p* < 0.001) band. The frontal cortex region is responsible for the higher cognitive functions (Frith and Dolan, 1996; María et al., 2010), providing an area for various networks to play out different scenarios (Mesulam, 2002). Yampolsky et al. (2012), suggested that the frontal cortex is vital for awareness and attention. The present study found that the LRTCs increased primarily in the frontal region after SCS, at both the delta and alpha bands. As higher cognitive functions are correlated with the prefrontal region, we hypothesized that SCS could affect these MCS functions by enhancing the temporal integration. The LRTCs also significantly increased in the occipital region at the theta band (*p* < 0.001). It has been suggested that the occipital cortex is involved in language processing (Bedny et al., 2011). Tosoni et al. (2015) have found that the occipital cortex is related to coherent visual motion and responding. Therefore, the significantly enhanced LRTCs in the occipital region after SCS indicate that SCS may impact MCS patients’ brain functions, such as language processing and visual functions.

## Limitations

This study has two limitations. First, the GDFAE indices had a range of 0.6–1 (from the pre-SCS to the post-SCS state). Krzeminski et al. (2017) found that the DFA was 0.9 in a wakened state and 0.6 under general anesthesia at the alpha oscillation in electrocorticographic data recordings. In our study, after the SCS, the DFA exponents of some patients passed 0.9, but these patients remained in MCS. We could not confirm whether the high DFA (> =0.9) meant that the patients had been conscious after the SCS. However, the relative changes in the GDFAE indices revealed the tendency for change in the complex brain system. Furthermore, an arbitrary quantification based on one parameter to assess the complex brain system is limited. Further studies should consider the multi-dimensional features to analyze the mechanism of the SCS (Kim et al., 2018). The multi-dimensional features should be considered to analyze the mechanism of the SCS in further studies (Kim et al., 2018). Second, as the brain activities of MCS patients have pathological oscillations, the dynamics of the system are different from those in a normal brain. The finding that LRTCs are correlated with consciousness has been established from studies of healthy brain tissue. Similar conclusions should be interpreted cautiously for MCS patients.

## Conclusion

The SCS showed a strong effect on EEG signals in patients with MCS whose long-range temporal integrations of brain activity had significantly increased (at low-frequency bands) in the frontal and occipital regions. Considering its close relationship with level of consciousness, we suggest that the GDFAE could serve as a new tool to explore the mechanisms of SCS in MCS patients.

## Author Contributions

ZL, JL, and YB designed this study. ZL collected the important background information. JL performed the statistical analysis. YB and JH carried out the study. YW and XX provided assistance for data acquisition. ZL and JL drafted the manuscript. YB and XL approved the final manuscript.

## Conflict of Interest Statement

The authors declare that the research was conducted in the absence of any commercial or financial relationships that could be construed as a potential conflict of interest.
